# Predicting conversion from mild cognitive impairment to Alzheimer's disease using brain ^1^H-MRS and volumetric changes: A two- year retrospective follow-up study

**DOI:** 10.1016/j.nicl.2019.101843

**Published:** 2019-04-30

**Authors:** Micaela Mitolo, Michelangelo Stanzani-Maserati, Sabina Capellari, Claudia Testa, Paola Rucci, Roberto Poda, Federico Oppi, Roberto Gallassi, Luisa Sambati, Giovanni Rizzo, Piero Parchi, Stefania Evangelisti, Lia Talozzi, Caterina Tonon, Raffaele Lodi, Rocco Liguori

**Affiliations:** a Department of Biomedical and NeuroMotor Sciences, Functional MR Unit, University of Bologna, Bologna, Italy; b IRCCS Istituto delle Scienze Neurologiche di Bologna, UOC Clinica Neurologica, Bologna, Italy; c Department of Biomedical and NeuroMotor Sciences, University of Bologna, Bologna, Italy; d Department of Physics and Astronomy, University of Bologna, Bologna, Italy; e Department of Biomedical and Neuromotor Sciences, Section of Hygiene and Biostatistics, University of Bologna, Bologna, Italy; f Department of Experimental, Diagnostic and Specialty Medicine, University of Bologna, Bologna, Italy; g IRCCS Istituto delle Scienze Neurologiche di Bologna, Diagnostica Funzionale Neuroradiologica, Bologna, Italy

**Keywords:** Mild cognitive impairment, MRI, ^1^H-MRS, Alzheimer's disease

## Abstract

This study investigated the ability of magnetic resonance spectroscopy (^1^H-MRS) of posterior cingulate cortex (PCC) and brain volumetry to predict the progression from mild cognitive impairment (MCI) to Alzheimer's Disease (AD) on the basis of clinical classification at 2 years follow-up. Thirty-eight MCI patients, eighteen healthy older adults and twenty-three AD patients were included in this study. All participants underwent a brain-MR protocol (1.5 T GE scanner) including high-resolution T1-weighted volumetric sequence (isotropic 1mm^3^). Voxel-wise differences in brain volumetry were evaluated using FreeSurfer software and all volumes were normalized by the total intracranial volume (TIV). Careful localization of ^1^H-MRS volume of PCC was performed and data were processed with the LCModel program. MCI patients underwent a complete neuropsychological assessment at baseline and were clinically re-evaluated after a mean of 28 months; twenty-six MCI patients (68.4%) converted to AD and twelve remained stable.

At baseline these two MCI subgroups did not differ in the global cognitive level (Mini Mental State Examination, MMSE) or in any of the other cognitive domains; the NAA/ mI ratio in the PCC was able to differentiate MCI converters from those MCI that did not develop AD (p = 0.022) with a level of accuracy (AUC area) of 0.779. A significantly reduced volume of parahippocampal gyrus (p = 0.010) and fusiform gyrus (p = 0.026) were found in the converter MCI subgroup compared to the stable MCI subgroup. The combined use of both N- acetyl-aspartate (NAA)/myo-Inositol (mI) ratio and volume of parahippocampal gyrus, increases the overall accuracy (AUC = 0.910) in predicting the conversion to AD two years before the development of clinical symptoms. Additional longitudinal studies with a broader representative sample of MCI patients and longer follow-up might be helpful to confirm these results and to elucidate the role of each parameter in predicting the possible progression to AD, and also to all the other non-AD dementia subtypes.

## Highlights

• Alterations of NAA/ml ratio in the PCC show high accuracy in predicting the progression to AD.
• Volume reduction of parahippocampal and fusiform gyrus are able to distinguish stable from converter MCI.
• The combined use of both NAA/mI ratio and volume of parahippocampal gyrus increases the accuracy.

## 1 Introduction

Mild Cognitive Impairment (MCI) is an intermediate clinical stage between the expected cognitive decline of normal aging and the very earliest features of dementia (Albert et al., 2011). Longitudinal studies provide evidence for different possible progression of MCI patients, ranging from the development of Alzheimer's Disease (AD) or non-AD dementias to the stabilization or even reversion of cognitive impairments (Mitchell and Shiri-Feshki, 2009). MCI is a very complex and highly nuanced clinical phenomenon with emerging evidence of the heterogeneity regarding the neurological, neuropsychological and underlying neuropathology (Petersen et al., 2009; Libon et al., 2010). The term “MCI due to AD” denotes a subgroup of patients with MCI with a high likelihood of underlying AD pathology, however the early distinction of these patients from those MCI that may present other causes of cognitive decline remains a big challenge. Today, there is converging belief that effective pharmacological and non-pharmacological treatment, such as cognitive stimulation, transcranial Direct Current Stimulation (tDCS) or repetitive Transcranial Magnetic Stimulation (rTMS) should focus on early stages of the disease (Freitas et al., 2011).

In clinical practice, a combination of clinical, neuropsychological and multimodal neuroimaging findings may offer at the MCI stage substantial information on the possible underlying pathology, thus leading to the early recognition of prodromal AD cases. In the last years, while a large body of literature focused on the progression from normal aging to MCI, thus on the early diagnosis of MCI, only fewer studies have investigated with advanced MRI techniques the specific features that may help predicting the progression from MCI to dementia. Distinguishing “MCI due to AD” from the other MCI subgroups is still a matter of debate.

Proton Magnetic resonance spectroscopy (^1^H-MRS) is an advanced non-invasive MR technique able to detect metabolic neurodegenerative changes by the quantification of N acetyl-aspartate (NAA), neuro-axonal marker, and myo-Inositol (mI), glial marker (Oz et al., 2014). ^1^H-MRS technique show tissue damage that may precede the evidence of atrophy on morphological imaging and the accuracy of this technique was demonstrated by the correlation of metabolic MR biomarkers with clinical and neuropathological severity in neurodegenerative disorders such as Alzheimer's disease (Kantarci et al., 2008). In addition, the MRS Consensus Group have indicated the ^1^H-MRS as a complementary tool to conventional MRI for diagnosing and monitoring disease progression and treatment response in neurodegenerative disorders (Oz et al., 2014).

Kantarci and colleagues reported that quantitative MRI and ^1^H-MRS, specifically hippocampal volume and NAA/mI of Posterior Cingulate cortex (PCC) are good predictors of MCI in cognitively normal older adults (Kantarci et al., 2013). In another recent study, Warangai and colleagues showed that cognitively normal elderly subjects with low NAA/mI might be at risk of progression to develop MCI (Waragai et al., 2017). All these findings highlight the role of these neuroradiological markers in predicting the development of neurodegenerative disorders in healthy subjects.

Furthermore, the PCC plays a key role in cognitive functioning, specifically in episodic memory retrieval and attention, and it is involved in maintaining the balance between internal and external thought (Leech and Sharp, 2014). Previous studies demonstrated that structural and functional abnormalities in this region are associated with cognitive impairments in neurodegenerative disorders (Buckner et al., 2005). The PCC is a highly connected and metabolically active brain region, therefore, a detailed understanding of the effects of neurodegeneration on the PCC is likely to be important, especially at the early stage.

The aim of our study was to investigate the ability of ^1^H-MRS of PCC and brain volumetry to predict the progression from MCI to AD on the basis of clinical classification at 2 year follow-up, by evaluating brain metabolites changes and reduction of specific brain volume regions at baseline.

## 2 Materials and methods

### 2.1 Participants

This retrospective study included 38 MCI patients (20 males; age, mean ± standard deviation = 73.9 ± 7.4 years) and 25 AD patients (15 males; age 70.8 ± 9.3 years). Eighteen healthy older volunteers (10 males; age 65.4 ± 9.5 years) without evidence of neurological, psychiatric or history of clinically significant diseases known to affect brain were also included. All patients were referred between 2009 and 2016 to the Functional MR Unit, S.Orsola-Malpighi Hospital, Bologna (IT), to perform brain MR investigation as part of the diagnostic workup. Controls were selected from the MR Functional Unit database of healthy volunteers (Ethical Committee approval Cod.: 120/2014, 7.10.2014).

Diagnosis were performed by neurologists experienced in neurodegenerative disorders, according to international criteria for MCI (Petersen, 2004) and AD (McKhann et al., 2011).

Clinical and neuropsychological data were collected from clinical records at baseline and after 24 months; the maximum interval between the baseline and the MR scan was three months. At follow-up, all MCI patients that evolved to Parkinson's disease, frontotemporal dementia, Lewy bodies disease or to any other neurodegenerative disorders different from AD were excluded from the study. Demographic and clinical features of cases and controls are summarized in Table 1. All subjects gave consent to personal data processing for research purposes and the protocol was approved by the local Ethical Committee (v. 1.0 April 2010).

**Table 1:** Demographic and clinical features of the study groups.

	Healthy controls (N = 18)	MCI non-converter (N = 12)	MCI converter (N = 26)	AD (N = 25)
Gender (M/F)	10/8	6/6	14/12	15/10
Age M (SD)	65.4 (9.5)	74 (8.3)	73.8 (7.2)	70.8 (9.3)
Follow-up in months M(SD)	-	29 (22.6)	27.2 (12.0)	-

### 2.2 Neuropsychological assessment

All MCI patients were administered the following standardized neuropsychological battery: Mini Mental State Examination (MMSE) (Folstein et al., 1975), a general screening test; Rey Auditory Verbal Learning Test (RAVLT) (Carlesimo et al., 1996) and visual memory test (Carlesimo et al., 1996), verbal memory tests; Analogies test (Gallassi et al., 2002), a verbal reasoning test; Verbal Associative Fluency Test (Carlesimo et al., 1996) and Category Words Fluency Test (Novelli et al., 1986), language tests. Furthermore, to explore executive function, spatial attention and visuo-constructive abilities, Stroop test (Caffarra et al., 2002), Barrage test (Gallassi et al., 2002), and the Copy Drawing test (Carlesimo et al., 1996) were administered. The severity of depression and anxiety symptoms was evaluated using Beck Depression Inventory (BDI) (Beck et al., 1961) and the State-Trait Anxiety Inventory (STAI X1 and STAI X2) (Spielberger, 1980).

### 2.3 MRI protocol acquisition

**Fig. 1:**
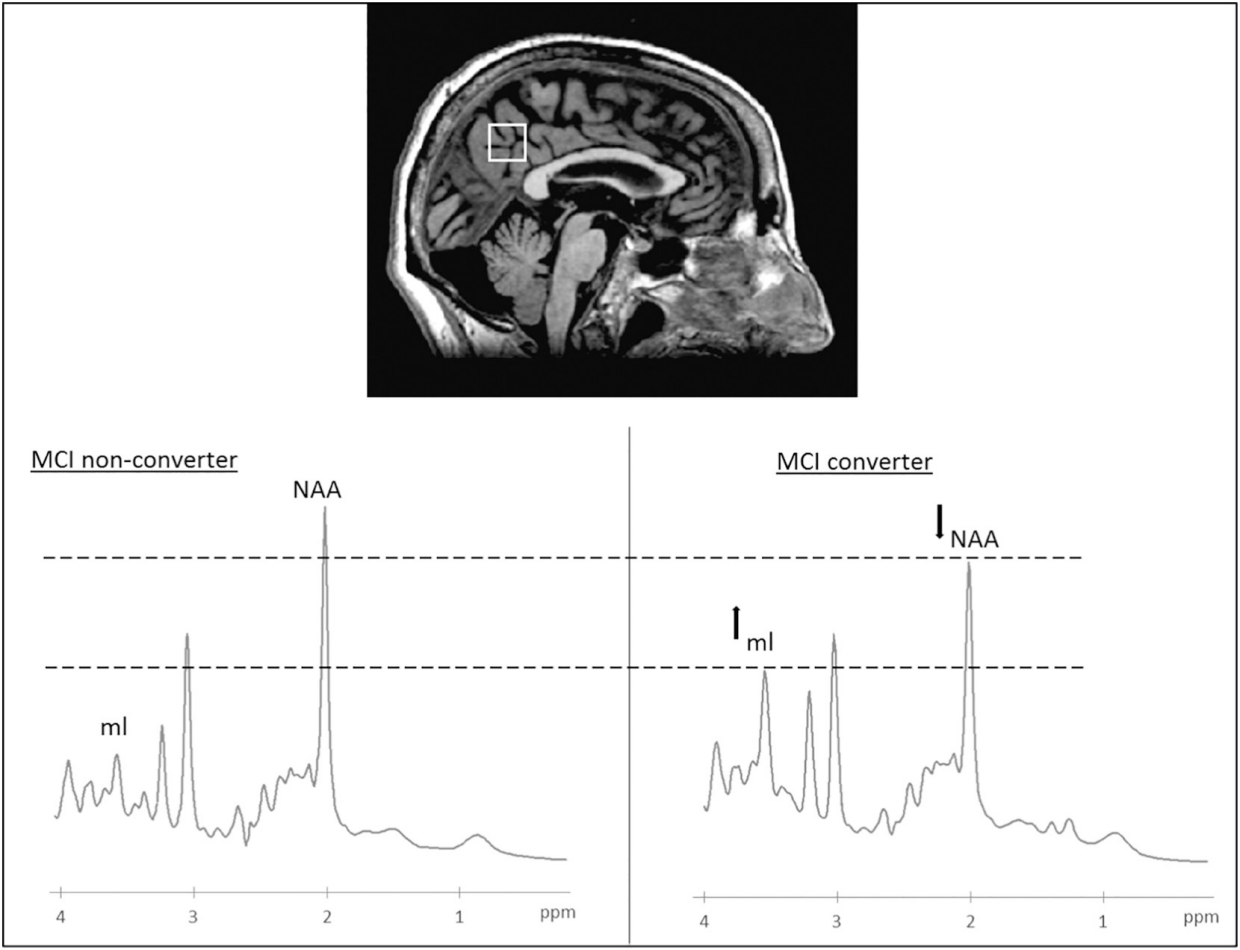
Above: example of the posterior cingulate cortex voxel localization projected onto sagittal plane of subject's own T1-wimage. Below: example of ^1^H-MR spectra indicating the resonances of interest (NAA: N-acetyl aspartate, ml: myo-Inositol) expressed as parts of million (ppm). Left: MCI non-converter, right: MCI-to-AD converter.

Brain MR studies were performed using a 1.5 Tesla GE Medical Systems Signa HDx 15 system equipped with a quadrature birdcage head coil. Structural imaging included axial FLAIR T2-weighted images (repetition time, TR = 8000 ms; inversion time, TI = 2000 ms; echo time, TE = 93.5 ms; 4 mm slice thickness with no inter-slice gap), FSE coronal T2-weighted images (TR = 7000 ms TE = 100 ms, 4 mm slice thickness) and 3D volumetric T1-weighted FSPGR images (TR = 12.5 ms, TE = 5.1 ms, TI = 600 ms, 25.6 cm^2^ FOV; 1 mm^3^ isotropic voxels). Single voxel ^1^H spectra were obtained from the posterior cingulate cortex (Volume Of Interest, VOI = 2.0 × 2.0 × 2.0 cm) (Fig. 1) using the three planes of high resolution 3D T1FSPGR sequence to optimise the localisation. Suppressed-water proton MR spectra were acquired using the PRESS localization sequence (PROBE) with TR = 4000 ms, TE = 35 ms, and averaging 128 FIDs for each acquisition (Lodi et al., 2009).The total acquisition scan time was about 40 min. Brain MR images obtained from each subject were visualized by an expert neuroradiologist (RL) in order to exclude other or significant abnormalities in patients and controls.

### 2.4 Volumetric analysis

Voxel-wise differences in brain volumetry were evaluated on 3D T1 FSPGR images using the freely available software FreeSurfer v5.3.0 (http://surfer.nmr.mgh.harvard.edu/) and all volumes were normalized by the total intracranial volume (TIV) of each participant.

### 2.5 Proton MR spectra analysis

The quality of each ^1^H MR spectrum was visually assessed by an expert physicist (ClT) blind to the clinical condition, according to standardized quality criteria (Oz et al., 2014). Peak areas of NAA + NAA glutamate (NAA), Cr + phospho-Cr (PCr), glycero-phospho Cho (GPC) + phospho Cho (PCh), and mI, were calculated using version 6.3 (http://www.lcmodel.com/) of the fitting program LCModel (Provencher, 2001). Automatic quantitation of localized in vivo 1H spectra with LCModel, a fully user-independent software, that analyzes spectra as a linear combination of complete model spectra of metabolite solutions in vitro. Metabolite content was expressed relative to Cr + PCr. NAA was also expressed relative to mI. The exclusion criteria for metabolite evaluation was an LCModel estimated fitting error >20%, this being a reliable indicator of poor quality spectra (Zanigni et al., 2015).

### 2.6 Statistical analysis

The normality of the distribution of all parameters was tested using Shapiro-Wilk test. The gender distribution was compared between groups using Pearson's χ^2^ -test. The non-parametric Mann-Whitney *U* test or Kruskal–Wallis test, followed by a Bonferroni post-hoc test for multiple comparisons, were used to compare neuropsychological data and quantitative MR parameters (all cortical and subcortical regions obtained with FreeSurfer) between groups. In order to determine the predictive accuracy of the parameters in discriminating MCI converters from non-converters, the non-parametric area under receiver operating characteristics (ROC) curve was calculated. Lastly, logistic regression analysis was used to estimate the probability of conversion to AD as a function of brain volumetry parameters and metabolite ratios. Furthermore, to analyse the association between metabolite ratios, brain volumetry and cognitive functions, Spearman's correlation coefficients were calculated between all variables. Statistical significance was set at p < 0.05 and all analyses were performed using IBM SPSS v.25 and Stata, version 15.

## 3 Results

### 3.1 Demographic, clinical and cognitive data

After a mean follow-up of 28 months, 26 MCI patients (68.4%) converted to AD, and 12 MCI remained stable. At baseline the two MCI subgroups did not differ in the global cognitive level (MMSE) or in any of the other cognitive domains (Table 1). Demographic and clinical characteristics of the study groups are summarized in Table 2. No brain MRI abnormalities were found in healthy controls and no patients showed brain lesions suggestive of secondary causes of neurological diseases.

**Table 2:** Neuropsychological data of MCI converter vs MCI stable at baseline (group comparison: Mann Whitney U test).

Cognitive tests	MCI converter M (SD)	MCI non-converter M (SD)	P value
MMSE	25.9 (2.7)	26.8 (2.4)	0.341
RAVLT - immediate	25.2 (7.3)	31.4 (10.3)	0.067
RAVLT - delayed	2.29 (2.9)	5.5 (3.5)	0.014
Visual memory	16.9 (3.7)	17.2 (3.4)	0.773
Analogies	13.7 (4.1)	15.0 (2.7)	0.336
Phonemic fluency	24 (10.6)	27.0 (9.7)	0.420
Semantic fluency	21.6 (7.3)	23.7 (6.1)	0.173
Stroop (time)	41.8 (20.9)	34.0 (10.9)	0.471
Barrage (time)	80.3 (39.9)	77.6 (26.7)	0.920
Barrage (error)	4.2 (5.9)	1.4 (1.2)	0.481
Copy drawing	9.5 (2.3)	9.4 (2.8)	0.959
BDI	12.9 (12.0)	12.0 (9.0)	0.967
STAI X1 (trait)	45.8 (9.6)	41.1 (9.1)	0.482
STAI X2 (state)	41.8 (11.3)	38.8 (9.9)	0.687

P < 0.003 (correction per multiple comparisons).

MMSE = Mini Mental State Examination; RAVLT = Rey Auditory Verbal Learning Test; BDI = Beck Depression Inventory; STAI = State-Trait Anxiety Inventory.

### 3.2 Brain volumetry

Compared with healthy controls, MCI and AD patients showed a widespread pattern of volume reduction in several temporo-parietal areas. One AD patient was excluded from this analysis due to suboptimal FreeSurfer brain MR parcellation.

When we split the MCI group into two subgroups the only parameters that were able at baseline to significantly discriminate converter MCI from stable MCI were the volume of parahippocampal gyrus and the volume of fusiform gyrus.

**Fig. 2:**
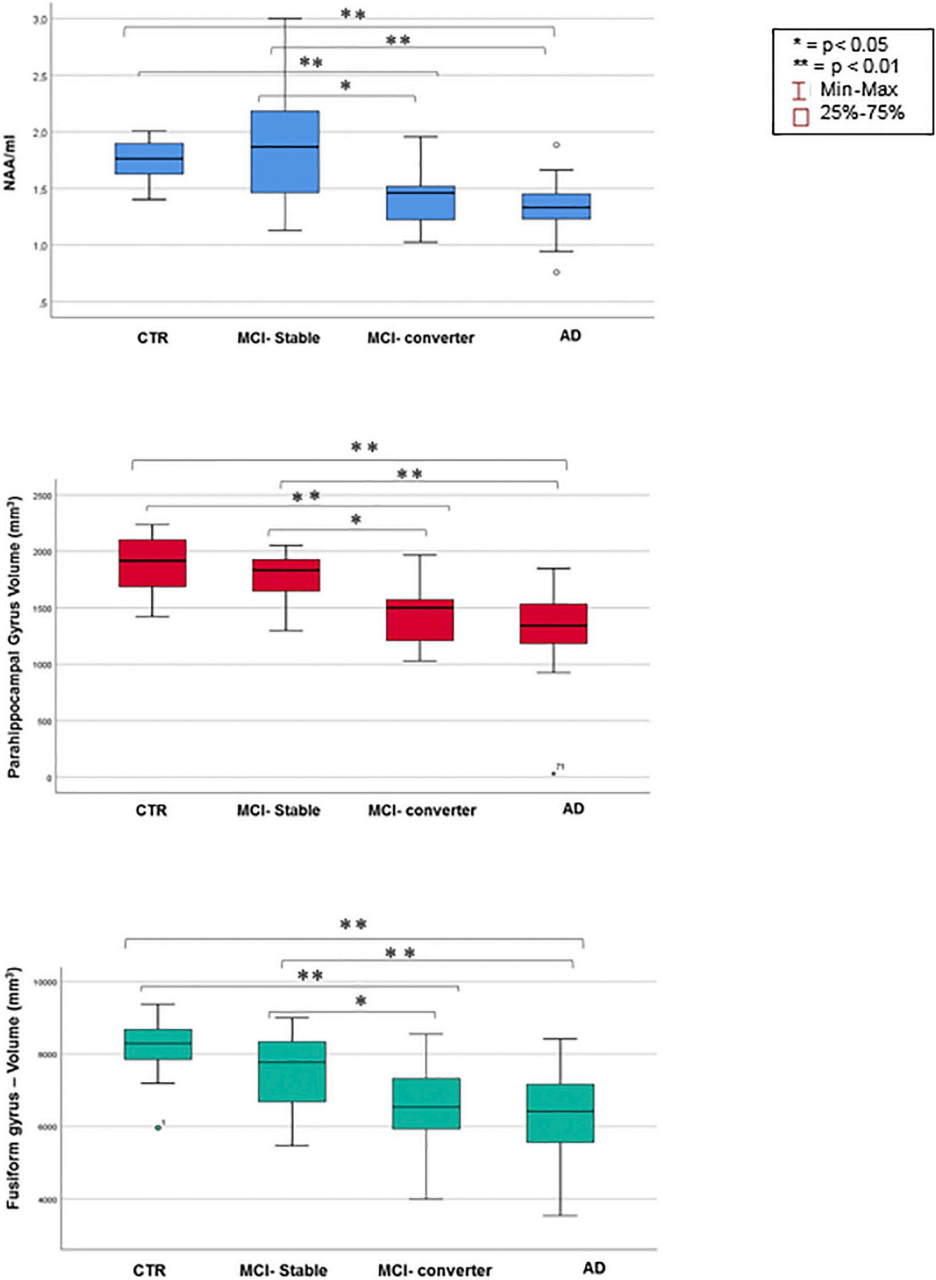
Boxplots showing the distribution of NAA/mI, parahippocampal volume and fusiform gyrus volume in healthy controls, MCI stable, MCI converter and AD patients.

Parahippocampal gyrus was also able to discriminate AD patients from healthy controls with a positive predictive value (PPV) of 91.3%; fusiform gyrus was able to discriminate both group with a PPV of 87%. Volumetric analysis showed similar volumes between stable MCI and healthy controls (parahippocampal gyrus mean 1771.17 ± 220.89 mm^3^ vs 1857.06 ± 254.75 mm^3^; fusiform gyrus mean 7496.58 ± 1170.45 mm^3^ vs 8202.44 ± 781.82 mm^3^). Similar volumes were also found between converter MCI and AD patients (parahippocampal gyrus mean 1448.08 ± 235.09 mm^3^ vs 1323.96 ± 349.98 mm^3^; fusiform gyrus mean 6600.46 ± 1071.01 mm^3^ vs 6421.42 ± 1235.61 mm^3^) (Fig. 2).

A significantly reduced volume of parahippocampal gyrus (p = 0.010) and fusiform gyrus (p = 0.026) were found in the converter MCI subgroup compared to the stable MCI subgroup.

**Fig. 3:**
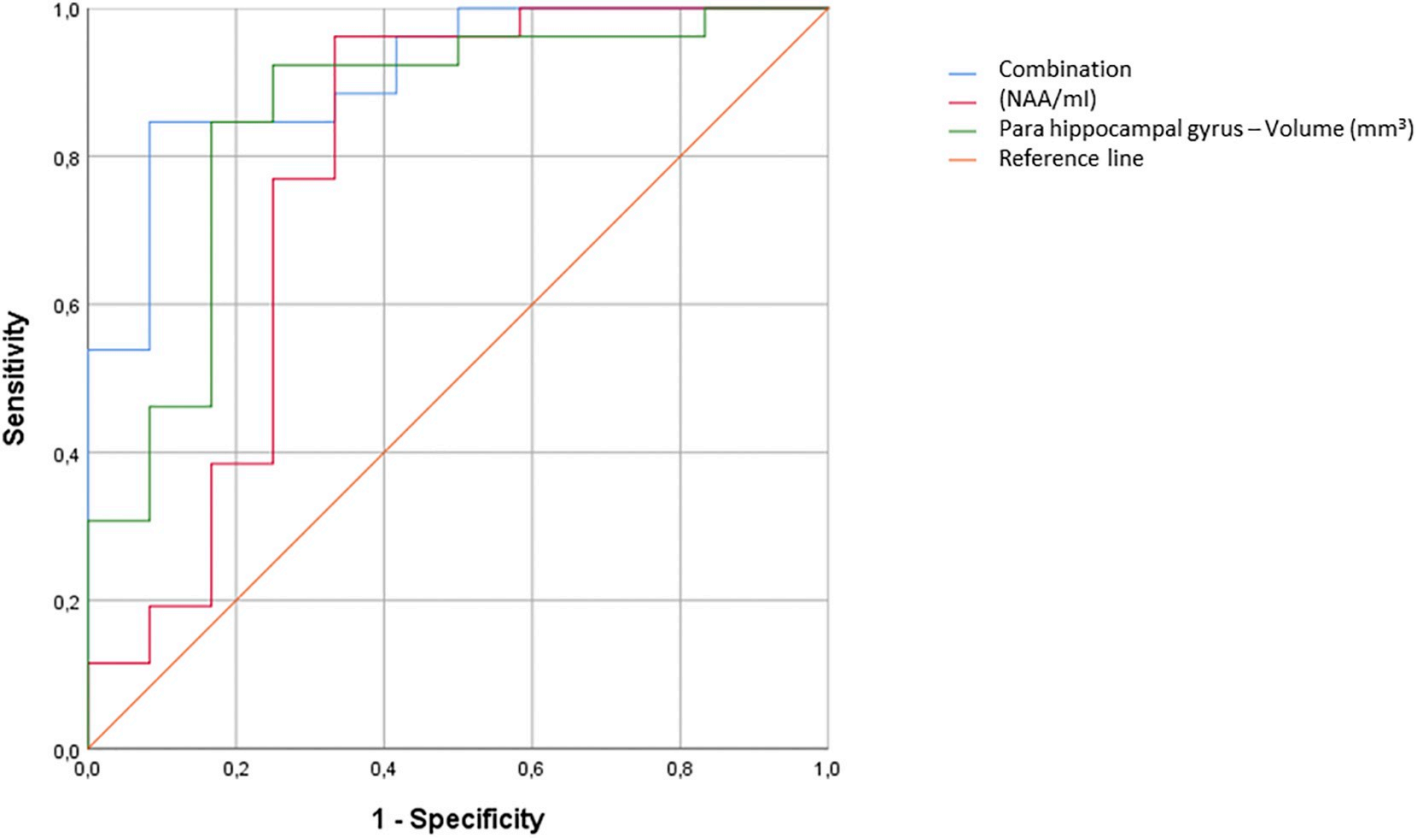
ROC curves for baseline NAA/ml, baseline brain volumetry (parahippocampal gyrus) and their combination as predictors of conversion from MCI to AD. NAA/mI: AUC=0.779; sensitivity 76.9%; specificity 75.0%; PPV 87.0%. Parahippocampal gyrus volume: AUC=0.853; sensitivity 84.6%; specificity 83.3%; PPV 91.7%. Combination of parameters: AUC=0.910; sensitivity 84.6%; specificity 91.7%; PPV 95.6%.

The areas under the ROC curve showed respectively a good accuracy for the parahippocampal gyrus (AUC = 0.853, 95% CI 0.711–0.994) in discriminating the two MCI subgroups and a lower accuracy for the fusiform gyrus (AUC = 0.705, 95% CI 0.506–0.904) (Fig. 3).

The optimal cut-off for the parahippocampal gyrus volume that balanced sensitivity and specificity in discriminating converters from non-converters was ≤1604mm^3^. At this cut-off the sensitivity was 84.6%, the specificity 83.3% and the PPV 91.7%.

For the fusiform gyrus the cut-off was ≤7132mm^3^, with a sensitivity of 73.1%, a specificity of 75.0%, and a PPV of 86.4%. It should be underscored that for both parameters, parahippocampal gyrus and fusiform gyrus volume, lower values predicted a higher probability of conversion to AD.

### 3.3 Proton MR spectroscopy of the PCC

The NAA/ mI ratio in the PCC differentiates healthy older adults (mean 1.76 ± 0.17) from MCI (mean 1.56 ± 0.38) (p = 0.011), but also MCI patients from AD (mean 1.32 ± 0.25) (p = 0.038). This value was able to discriminate AD patients from healthy controls with a PPV of 95%. Furthermore, this metabolites ratio was also able to discriminate at baseline MCI converters (mean 1.42 ± 0.23) from those MCI that did not develop AD (mean 1.85 ± 0.47) (p = 0.022). ROC curve analysis showed an overall accuracy of 0.779 (95% CI 0.586–0.972). The optimal cut-off was ≤1.52, with a sensitivity of 76.9%, a specificity of 75.0% and a PPV of 87% (Fig. 3).

### 3.4 Predictive accuracy using combinations of parameters

We carried out a further analysis to determine whether an incremental accuracy could be achieved by combining the parameters. *Z*-scores were calculated for each parameter and a binary logistic stepwise regression analysis was performed. The predicted probability of conversion obtained from the model using parahippocampal gyrus and NAA/mI (fusiform gyrus was removed because it was no longer significant), was used in a ROC analysis. The overall accuracy (AUC area) in discriminating the two MCI subgroups obtained by combining parahippocampal gyrus and NAA/mI increased to 0.910 (95% CI 0.815–1.000). The optimal cut-off was ≤0.69, with a sensitivity of 84.6%, a specificity of 91.7% and a PPV of 95.6% (Fig. 3).

The calculator of the Supplementary Table provides the probability of conversion to AD estimated using logistic regression models (Supplementary material).

### 3.5 Correlation analysis

A significant correlation was found between the volume of the parahippocampal gyrus and two measures of memory, specifically verbal short-term memory (r = 0.35, P = 0.035) and verbal long-term memory (r = 0.34, P = 0.039). The scores obtained for the short-term memory task also correlated with the fusiform gyrus volume (r = 0.34, P = 0.039). No significant correlations were found between metabolite ratios and cognitive functioning.

## 4 Discussion

The present study highlights that alterations of metabolite levels of PCC, specifically NAA/ml, showed high accuracy not only in the discrimination between healthy controls, MCI and AD, but also in predicting the possible progression to AD in a group of MCI patients. These findings are in line with previous cross-sectional studies that showed increased level of mI as an early marker of neurodegenerative changes in patients with MCI and decreased level of NAA and further elevated mI in AD patients (Oz et al., 2014; Miller et al., 1993).

Interestingly, volume reduction of parahippocampal gyrus and fusiform gyrus were also able to discriminate at baseline stable MCI from those MCI that subsequently converted to AD and these volumes are associated with memory deficits in the whole sample. Echavarri and colleagues suggested that parahippocampal volume discriminates better than hippocampus between cases of healthy aging, MCI, and AD, in particular, in the early phase of the disease (Echávarri et al., 2011). Furthermore, these results also confirm Li and colleagues study that demonstrated the role and the involvement of parahippocampal cortex in memory encoding and retrieval (Li et al., 2010). Previous studies showed that the hippocampal subregions uniquely contribute to cognitive processes (Kesner et al., 2004), and are differentially affected by AD pathology over time (Hara et al., 2013). The hippocampus is comprised of several subfields including the dentate gyrus, subiculum, and cornu ammonis subfields 1–4 (CA1, CA2, CA3, and CA4) (Duvernoy, 2005). Some studies have reported subregion-specific hippocampal atrophy related to the presence and spread of neurofibrillary tangles in the hippocampal structures (Greene and Killiany, 2012; Hara et al., 2013). Recently, Blanken and colleagues demonstrated that hippocampal atrophy was strongly associated with AD diagnosis and neuronal loss (Blanken et al., 2017). Specifically, the most pronounced associations spanned the locations corresponding to the CA1 and subiculum subfields, which are thought to be the earliest and most severely affected subfields in AD (Schönheit et al., 2004). Authors conclude that atrophy in these two subfields is most predictive of future conversion from healthy controls to MCI and from MCI to dementia (Apostolova et al., 2006; Apostolova et al., 2010).

The present study shows that parahippocampal gyrus, a structure adjacent to the subiculum, can be a valuable marker of early neurodegeneration. Logistic regression models using the volume of parahippocampal gyrus, estimated in a group of MCI patients the probability of conversion to AD with a PPV of 91.7%. These data indicate that once AD-related parahippocampal atrophy is prominent enough in MCI patients, further cognitive decline and loss of functional independence is imminent. In addition, alterations of NAA/mI in the PCC are also likely to be an important finding in this study, estimating the probability of conversion to AD with a PPV of 87%. The PCC forms a central node in the default mode network (DMN) of the brain, and previous studies demonstrated that the connections between the PCC and the hippocampus areas are altered at a very early stage of AD (Zhou et al., 2008). Recently, Ward and colleagues showed that resting-state connectivity between the hippocampus and PCC is indirect and mediated by the parahippocampal gyrus (Ward et al., 2014). Authors highlight that parahippocampus, rather than the hippocampus, is the primary hub of the DMN in the medial temporal lobe (MTL), therefore, it may prove to be a particularly promising biomarker of early Alzheimer's disease-related network dysfunction. Based on current models of biomarkers of AD pathophysiology (Jack Jr et al., 2013) our results confirm that ^1^H-MRS and MRI-derived marker of neurodegeneration are useful in short-term prognosis of MCI patients. Moreover, we demonstrates that the combined use of both NAA/mI and volume of parahippocampal gyrus, increases the accuracy in predicting the conversion to AD. These results further corroborate those from emerging post-mortem studies that explore this temporal dissociation between the neuropathological and clinical changes (Suemoto et al., 2017).

However, our study is limited by its small sample size, therefore, additional longitudinal studies with a larger representative sample of MCI patients and longer follow-ups might be helpful to confirm these results and to elucidate the role of each parameter in predicting the possible progression to AD, but also to all the other non-AD dementia subtypes. Future evidence is needed to better determine whether these findings are generalizable to clinical practice.

## 5 Conclusion

Predicting the possible evolution from the prodromal MCI stage to dementia is a big challenge for both research and clinical practice. Conversion to dementia is a primary outcome measure in interventional clinical trials and predictors of time to conversion may serve as ‘surrogate endpoints’. Furthermore, predictors of AD are also of pivotal importance in clinical practice by assisting clinicians during patient work-up.

## Funding

This study has been supported by the Italian Ministry of Health (#GR-2013-02358026).

## Disclosure statement

Rocco Liguori has acted as a paid advisor to Biogen, Sanofi- Genzyme, Argon Healthcare s.r.l., Editree Eventi s.r.l.; has received Lecture funding from Dynamicom Education, SIMG Service, Adnkronos Salute unipersonale s.r.l., DOC Congress s.r.l., First Class s.r.l., Fondazione Società Italiana di Neurologia and has acted as a paid consultant to Alfasigma and Amicus Therapeutics s.r.l. outside of the research carried out in this work.

The other authors have no conflict of interest to disclose.

## Appendix A Supplementary data

Supplementary material Image 1
